# Dr. Himmatrao Bawaskar: Transforming Rural Healthcare Through Scientific Inquiry and Advocacy

**DOI:** 10.7759/cureus.62006

**Published:** 2024-06-09

**Authors:** Umesh Kawalkar, Bhagwat Rajput, Amar Mankar, Priti Kogade

**Affiliations:** 1 Community Medicine, Government Medical College (GMC) Akola, Akola, IND; 2 Psychiatry, World College of Medical Sciences Research and Hospital (WCMSRH), Gurawar, IND; 3 Community Medicine, Datta Meghe Institute of Higher Education & Research, Wardha, IND; 4 National Tobacco Control Program, Public Health Department, Akola, IND

**Keywords:** padma shree, maharastra, biography, medical codes of ethics, snake-bite, prazosin, scorpion envenomation, historical vignette

## Abstract

Dr. Himmatrao Bawaskar, a distinguished figure in Indian healthcare, has made significant contributions to medical research and public health, particularly in rural areas. Born in 1951 in Maharashtra, his journey from a rural upbringing to receiving one of the highest civilian awards of the Government of India, the Padma Shri, reflects his dedication to the field of medicine and public health. Dr. Bawaskar's groundbreaking research on scorpion stings, notably the use of prazosin, has revolutionized treatment protocols, significantly reducing mortality rates. Beyond scorpion stings, his work spans diverse medical areas, including snake bites and cardiovascular diseases. Moreover, Dr. Bawaskar's advocacy for ethical practices and healthcare reform underscores his commitment to improving healthcare outcomes. His legacy serves as an inspiration for future generations of healthcare professionals and policymakers, emphasizing the transformative power of dedication, compassion, and scientific inquiry in addressing critical healthcare challenges.

## Introduction and background

**Figure 1:**
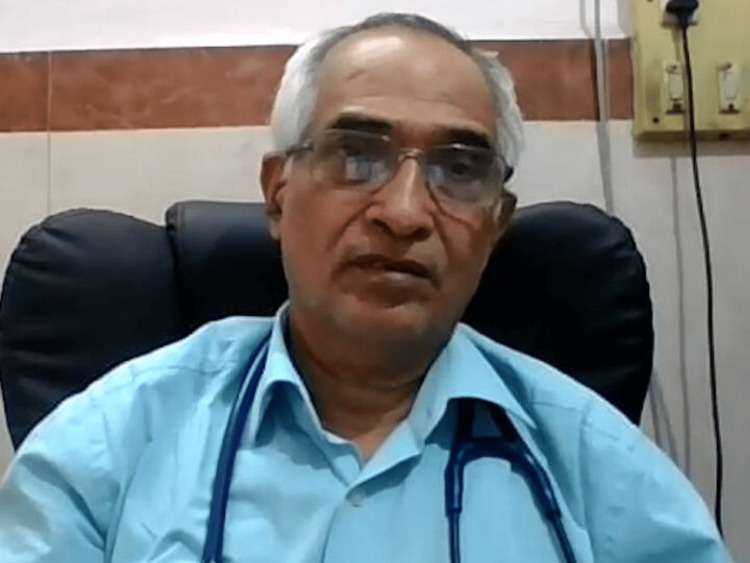
Dr. Himmatrao Bawaskar Permission to publish the photograph has been obtained from Dr. Himmatrao Bawaskar

Dr. Himmatrao Bawaskar (Figure 1), an esteemed Indian physician, has made significant contributions to medical research, particularly in the realm of treating scorpion stings. Born on March 3, 1951, in Dehed village (now in Bhokardan, Jalna), Maharashtra, India, his journey from humble beginnings to becoming a Padma Shri awardee showcases his dedication and perseverance in the field of medicine. Dr. Bawaskar's educational journey began with hardships as he navigated through challenges like class- and caste-based discrimination during his college days at Government Medical College and Hospital, Nagpur, India. Despite facing obstacles, he pursued his passion for medicine and completed his MD from the B. J. Medical College, Pune, India, in 1981.

His career path led him to serve at a government-funded primary health center in Birwadi, Raigad district, Maharastra, in 1976. It was here that he encountered the alarming fatality rates caused by scorpion stings, particularly affecting children. Witnessing this, he embarked on a mission to find effective treatments, leading to ground-breaking research published in prestigious medical journals. Dr. Bawaskar's research not only revolutionized the treatment of scorpion stings but also extended to diverse medical areas such as snake bites, cardiovascular diseases, and hypothyroidism. His relentless pursuit of knowledge and solutions has significantly impacted public health, particularly in rural areas of Maharashtra [1,2].

Moreover, Dr. Bawaskar's advocacy extends beyond medical research. He has been vocal about ethical practices, corruption in the medical field, public healthcare issues, violence against doctors, and the state of medical research in India. Through his writings and opinions, he continues to advocate for improvements in the healthcare system and medical ethics. Dr. Bawaskar's remarkable journey from a rural village to becoming a renowned physician exemplifies the transformative power of dedication, compassion, and scientific inquiry in addressing critical healthcare challenges [1].

## Review

Early life and career

Dr. Himmatrao Bawaskar was born in a small village in rural Maharashtra in the early 1950s, into a poor farming family. His father, despite being illiterate, believed that education was the key to overcoming poverty and was determined to educate his children, earning the nickname "barrister." Himmatrao, aptly named for his courage (*Himmat* (Hindi): courage), faced numerous hardships from a young age. To support his education, he worked various odd jobs, including in fields, hotels, temples, bookshops, chemist shops, and brick kilns. His perseverance led him to enroll at the Government Medical College in Nagpur, where he pursued an MBBS (Bachelor of Medicine, Bachelor of Surgery) degree. Despite feeling out of place and lacking guidance, he relied on his hard work and deep study. However, he also struggled with depression during his college years, which affected his confidence. It was only after completing his degree and undergoing prolonged treatment that he overcame these challenges [1].

After earning his MBBS degree, Himmatrao's strong ties to his rural roots influenced his decision to work as a Medical Officer at a Primary Health Centre (PHC). He chose to serve in underserved areas and managed to secure a position at a small PHC in the coastal district of Raigad, overcoming significant bureaucratic obstacles. This marked the beginning of his impactful career in rural healthcare. In Raigad, Dr. Bawaskar encountered the problem of scorpion stings, which were common and often fatal in the region. Motivated to find a scientific solution, he meticulously studied the cases and developed effective treatment methods, significantly improving patient outcomes. His dedication and innovative approach to treating scorpion stings made a lasting impact on rural healthcare in Maharashtra [1,3].

Revolutionizing scorpion sting treatment with prazosin

Despite facing significant issues like corruption, unclean premises, and unmotivated physicians at the PHC, he chose to work there with a strong personal commitment. He gradually reformed the PHC, establishing himself as a dedicated and skilled doctor. During this time, he encountered the problem of scorpion stings, which were surprisingly common and often fatal in the area. Local superstitions exacerbated the problem by discouraging medical treatment. Motivated to find a scientific solution, he collected data from various sources but found it unreliable. He then admitted all scorpion sting cases to study them directly, using limited resources like a stethoscope and a sphygmomanometer. Through careful observation and meticulous record-keeping, he identified pulmonary edema as the immediate cause of death. Traditional treatments proved ineffective, so he shared his findings with the Haffkine Institute, leading to a publication in *The Lancet* in 1978 [3].

Recognizing the need for further education, he pursued an MD in Medicine at B.J. Medical College, Pune, where he advanced his clinical skills. He prepared a detailed paper on 51 scorpion sting cases, initially rejected by an Indian journal but later accepted by *The Lancet* in 1982 [1,3]. The paper outlined the diagnostic signs and symptoms of red scorpion stings [4]. Observing a fatal scorpion sting case in a well-equipped hospital, he questioned the effectiveness of available treatments in less equipped settings like PHCs. He discovered that heart failure from scorpion stings resembled refractory heart failure and successfully treated many cases using sodium nitroprusside [1,3].

After earning his MD degree, Dr. Himmatrao Bawaskar, along with his wife Dr. Pramodini Bawaskar, returned to Konkan to find the same health issues persisting. In October 1983, they treated an eight-year-old child with severe scorpion sting symptoms, including pulmonary edema. Dr. Bawaskar, with the father's consent, used nitroprusside, a risky drug, to help the child's heart. He monitored the child closely, administering the drug drop by drop. After four hours, the child began to recover. Despite receiving news of his father's death during this time, Dr. Bawaskar chose to stay with his patient, considering it a tribute to his father's belief in his work [3].

News of the successful treatment spread quickly, and many patients from Konkan sought help. In one month, Dr. Bawaskar treated 65 patients with pulmonary edema. However, because nitroprusside was dangerous and required careful monitoring, he searched for a safer alternative. He found prazosin, an alpha-blocker, effective for conditions caused by excessive catecholamines, similar to scorpion venom effects. In 1984, he treated 126 patients with prazosin, all of whom survived [3]. His paper on this treatment was published in *The Lancet* in 1986, gaining international recognition [5]. Dr. Himmatrao Bawaskar's work gained international recognition as the success of prazosin in treating scorpion stings was replicated worldwide. His next goal was to spread this treatment among doctors in his home country. He organized seminars, communicated regularly with other doctors, and distributed academic materials on the subject. As a result, the case fatality rate from scorpion stings dropped from 40% to less than 1%. He continued to improve and standardize treatment protocols, publishing his findings in prestigious journals. Remarkably, all his research was self-funded, demonstrating that passion and dedication can drive impactful research without the need for extensive resources [1,3].

Dr. Bawaskar is now considered an international authority on scorpion stings and has authored two chapters in the Association of Physicians of India (API) Textbook of Medicine and prazosin as a treatment for scorpion stings has been included in this standard textbook of medicine [6]. In 2011, a randomized trial by Dr. Himmatrao Bawaskar and Dr. Pramodini Bawaskar, comparing the efficacy of scorpion antivenom combined with prazosin versus prazosin alone, was published in the British Medical Journal (BMJ) [7]. In the same issue of the BMJ, there was an editorial by Mills and Ford highlighting the significance of research in resource-poor settings, with reference to Dr. Bawaskar's trial [8]. Dr. Bawaskar's dedication and rigorous clinical observation methods have set a high standard for research aimed at improving human health.

Dr. Bawaskar's groundbreaking research conducted at a PHC underscores the vital role of local healthcare facilities in addressing community health challenges. His work exemplifies how even with limited resources, PHCs can serve as hubs for innovative research and effective interventions. By focusing on prevalent health issues within the community and leveraging his clinical expertise, Dr. Bawaskar demonstrated that significant advancements in medical knowledge and treatment outcomes can be achieved at the grassroots level. His success emphasizes the importance of investing in and empowering PHCs as essential pillars of healthcare delivery, particularly in underserved areas where they can make a profound impact on public health.

Community need-based research

Dr. Himmatrao Bawaskar, assisted by Dr. Pramodini Bawaskar, now operates a hospital in Maharashtra where they provide primary intensive care to patients. Their commitment to hands-on patient care reflects their dedication to serving their community [3]. Dr. Bawaskar's research endeavors have been inspired by the health challenges prevalent in rural areas. For instance, he explored thrombolytic therapy for acute myocardial infarction (AMI) in rural settings, prompted by the high prevalence of chronic diseases and coronary heart disease risk factors [9]. Additionally, Dr. Bawaskar's research extends to chronic renal failure [10], dental fluorosis [11], hypothyroidism suspicion scores [12], and even the potential therapeutic application of scorpion venom for Brugada syndrome [13]. His observations on snakebites have led to improved diagnosis and treatment strategies, particularly for Krait and Russel's viper bites, highlighting the importance of early intervention to prevent complications like acute renal failure [14]. Furthermore, Dr. Bawaskar's investigation into increased lead levels in petrol pump workers, idol painters, garage workers, etc. exemplifies his commitment to addressing health hazards in various occupational settings [15].

Dr. Bawaskar's research is rooted in real-world experiences and driven by a genuine desire to improve human health, rather than commercial interests. His advocacy for ethical medical practices, HIV-related issues, and community-based research underscores his commitment to advancing healthcare with integrity. With over 60 publications in prestigious journals and numerous speaking engagements at national and international conferences, his journey from humble beginnings to becoming an internationally recognized authority exemplifies the transformative power of dedication and compassion in addressing critical healthcare challenges.

## Conclusions

Dr. Himmatrao Bawaskar's exceptional contributions to medical research and public health have left an indelible mark on the landscape of Indian healthcare. His research was conducted at a PHC with limited resources. His work exemplifies how even with limited resources, PHCs can serve as hubs for innovative research and effective interventions. His groundbreaking research on scorpion envenomation, coupled with his relentless advocacy for ethical practices and healthcare reform, stands as a testament to his unwavering commitment to improving healthcare outcomes. Dr. Bawaskar's legacy serves as an enduring source of inspiration for healthcare professionals and policymakers, resonating with the transformative potential of dedication, compassion, and scientific inquiry. As we contemplate his lasting impact, we are reminded of the profound influence individuals like Dr. Bawaskar wield in addressing critical healthcare challenges and enhancing the well-being of millions, particularly in rural and underserved communities.

## References

[1] Bawaskar Himmatrao (2016). Barrister's Brat (Autobiography). https://www.bookganga.com/ebooks/Books/details/5747434729138651365.

[2] (2024). Padma Awards 2022 Anounced. https://pib.gov.in/PressReleasePage.aspx?PRID=1792640.

[3] Kale AA (2012). A crusade against scorpion sting: life and works of Dr. Himmatrao bawaskar. J Family Med Prim Care.

[4] Bawaskar H (1982). Diagnostic cardiac premonitory signs and symptoms of red scorpion sting. Lancet.

[5] Bawaskar HS, Bawaskar PH (1986). Prazosin in management of cardiovascular manifestations of scorpion sting. Lancet.

[6] (2015). API Texbook of Medicine. https://www.google.co.in/books/edition/API_Textbook_of_Medicine_Volume_I_II/z39GCgAAQBAJ?hl=en&gbpv=0.

[7] Bawaskar HS, Bawaskar PH (2011). Efficacy and safety of scorpion antivenom plus prazosin compared with prazosin alone for venomous scorpion (Mesobuthus tamulus) sting: randomised open label clinical trial. BMJ.

[8] Mills EJ, Ford N (2011). Research into scorpion stings. BMJ.

[9] Bawaskar HS, Bawaskar PH, Bawaskar PH (2019). Preintensive care: thrombolytic (streptokinase or tenecteplase) in ST elevated acute myocardial infarction at peripheral hospital. J Family Med Prim Care.

[10] Bawaskar HS, Bawaskar PH, Bawaskar PH (2010). Chronic renal failure associated with heavy metal contamination of drinking water: a clinical report from a small village in Maharashtra. Clin Toxicol (Phila).

[11] Bawaskar HS, Bawaskar PH (2006). Endemic fluorosis in an isolated village in western Maharashtra, India. Trop Doct.

[12] Bawaskar H (1998). Premonitory diagnostic signs and symptoms (scores) of thyroid hypofunction: a clinical observation. J Assoc Physicians India.

[13] Bawaskar H, Bawaskar PH, Bawaskar Bawaskar, PH PH (2022). Stung to the heart. J Am Coll Cardiol Case Rep.

[14] Bawaskar HS, Bawaskar PH (2020). Blood lead level among fuel station workers, Ganesh idol painters, persons with routine daily application lead containing black pigment to eyes and Garage workers. J Family Med Prim Care.

[15] Bawaskar HS, Bawaskar PH (2002). Profile of snakebite envenoming in western Maharashtra, India. Trans R Soc Trop Med Hyg.

